# Computational prediction of splicing regulatory elements shared by *Tetrapoda *organisms

**DOI:** 10.1186/1471-2164-10-508

**Published:** 2009-11-04

**Authors:** Alexander Churbanov, Igor Vořechovský, Chindo Hicks

**Affiliations:** 1 New Mexico State University, Biology Dept., MSC 3AF, PO Box 30001, Las Cruces, NM 88003, USA; 2 University of Southampton, Southampton University Hospital, MP808, Tremona Road, Southampton SO16 6YD, UK; 3 Loyola University Medical Center, 2160 S. First Ave., Maywood, IL 60153, USA

## Abstract

**Background:**

Auxiliary splicing sequences play an important role in ensuring accurate and efficient splicing by promoting or repressing recognition of authentic splice sites. These *cis-*acting motifs have been termed splicing enhancers and silencers and are located both in introns and exons. They co-evolved into an intricate splicing code together with additional functional constraints, such as tissue-specific and alternative splicing patterns. We used orthologous exons extracted from the University of California Santa Cruz multiple genome alignments of human and 22 *Tetrapoda *organisms to predict candidate enhancers and silencers that have reproducible and statistically significant bias towards annotated exonic boundaries.

**Results:**

A total of 2,546 *Tetrapoda *enhancers and silencers were clustered into 15 putative core motifs based on their Markov properties. Most of these elements have been identified previously, but 118 putative silencers and 260 enhancers (~15%) were novel. Examination of previously published experimental data for the presence of predicted elements showed that their mutations in 21/23 (91.3%) cases altered the splicing pattern as expected. Predicted intronic motifs flanking 3' and 5' splice sites had higher evolutionary conservation than other sequences within intronic flanks and the intronic enhancers were markedly differed between 3' and 5' intronic flanks.

**Conclusion:**

Difference in intronic enhancers supporting 5' and 3' splice sites suggests an independent splicing commitment for neighboring exons. Increased evolutionary conservation for ISEs/ISSs within intronic flanks and effect of modulation of predicted elements on splicing suggest functional significance of found elements in splicing regulation. Most of the elements identified were shown to have direct implications in human splicing and therefore could be useful for building computational splicing models in biomedical research.

## Background

Eukaryotic genes contain intervening sequences or introns that need to be removed from precursor messenger RNA (pre-mRNA) in a complex process termed splicing. During pre-mRNA splicing, relatively short exonic sequences are recognized by spliceosome, a large RNA-protein complex. During splicing, introns are removed and exons are joined together to form mature RNA. In addition to splice site (SS) signals at the exonic 5' and 3' ends, accurate discrimination of exons and introns requires additional auxiliary elements [1-3]. These conserved but degenerate motifs have been termed exonic (ESEs) and intronic (ISEs) splicing enhancers and exonic (ESSs) and intronic (ISSs) splicing silencers that activate or repress splicing, respectively. These elements are thought to bind splicing regulatory factors, including the serine/arginine-rich (SR) proteins and the heterogeneous nuclear ribonucleoproteins [1]. Consistent with this concept, splicing regulatory motifs were shown to associate with a single stranded conformation that is more accessible to protein-RNA interactions [2]. Combinatorial interaction of splicing factors bound by these motifs is important for both constitutive and alternative splicing of pre-mRNAs because they contribute to the regulation of gene expression and proteomic diversity across higher eukaryotes [3-6].

Several systematic computational approaches and *in vivo *or *in vitro *selection methods have been employed to identify these motifs in the genomic sequences. For example, the RESCUE-ESE (Relative Enhancer and Silencer Classification by Unanimous Enrichment), a computational approach used in conjunction with experimental validation, predicted specific hexanucleotide sequences as candidate ESEs based on significantly higher frequency of occurrence in exons than in introns and also significantly higher frequency in exons with weak SSs than in exons with strong SSs [7]. The number of putative exonic enhancer and silencer octamers were computationally identified by their enrichment in internal non-coding exons versus unspliced pseudoexons and 5' untranslated regions of transcripts in intronless genes [8]. A cell-based fluorescence-activated screen (FAS), an *in vivo *splicing reporter system was used to identify ESSs that demonstrated consistent silencing results in a splicing reporter construct [9]. Evolutionary conserved intronic splicing regulatory elements were found by considering intronic boundaries surrounding orthologous exons in *Homo sapiens*, *Canis familiaris*, *Rattus norvegicus *and *Mus musculus *obtained from UCSC genome-wide multiple alignments [10]. Putative splicing regulatory sequences were reported based on evolutionary conserved wobble positions between human and mouse orthologous exons, along with overabundance of sequence motifs compared to their random expectation [11]. Exonic and intronic elements have also been predicted based on strand asymmetry [12]. Neighborhood Inference (NI) approach predicted ESEs and ESSs with activity in regulating biochemical processes based on the local density of known sites in sequence space [13]. Finally, a recent study based on deep re-sequencing of human transcriptome [14] uncovered a new repertoire of plausible intronic hexamers supporting the tissue-specific splicing events.

A large fraction of spliceosomal components are highly conserved across eukaryotes, including *Tetrapoda *(four-footed) organisms [1,6,15-17], where the genes encoding well-known RNA binding proteins involved in splicing regulation are enriched with ultraconserved elements [18]. Three quarters of RESCUE-ESEs are shared between humans and mice [17]. Most of the human RESCUE-ESEs [7] have a pronounced bias towards exonic boundaries in more distantly related vertebrate organisms [17]. A number of experimental reports showed that genes from distantly related *Tetrapoda *organisms were correctly expressed and post-transcriptionally modified in transgenic animals [19,20]. These observations suggest that splicing regulatory motifs shared by tetrapods may further enrich known elements for functionally important sequences. However, no systematic studies have been carried out.

In this work, we predict an extensive set of *cis*-acting elements identified in a large set of *Tetrapoda *exons and characterize their overlap with previously identified silencers/enhancers. Unlike in previous methods, we did not restrict the size of ESE/ISE/ESS/ISSs oligomers unless they are longer than 8 nt. Our prediction is based on the assumption that auxiliary splicing elements have pronounced statistically significant density increase/decrease towards the exonic boundaries compared to the deep intronic or exonic sequences. This assumption allows using the identified elements to improve performance of splicing prediction methods. Predicted ISEs/ISSs close to the annotated exons were examined for increased evolutionary conservation as compared to oligos with no predicted functionality. Finally, we investigated association of the elements placed in context with the single-stranded configuration of local pre-mRNA structure.

## Results and Discussion

### Identification of splicing regulatory elements in tetrapods

Using 2,333,379 extended *Tetrapoda *exons, we predicted 2,546 unique splicing regulatory elements that have statistically significant density increase/decrease in the vicinity of SS compared to the deep intronic or exonic sequences. A total of 75 ESEs/ESSs and 1,846 ISEs/ISSs were found to support 3'SS, whereas 54 ESEs/ESSs and 652 ISEs/ISSs were found to influence 5'SS. Clusters of predicted elements could be found in [see Additional File 1 Section 4].

In primates, we predicted a total of 95 5'SS-related and 157 3'SS-related ISEs/ISSs [see Additional File 1 Section 5], whereas in the outgroup [see Subsection *Collection and validation of Tetrapoda exons*] we have found 88 5'SS-related and 330 3'SS-related ISEs/ISSs [see Additional File 1 Section 6]. Among the predicted elements for the primates and outgroup 21 5'SS ISEs/ISSs and 82 3'SS ISEs/ISSs were common between two clades. Splicing regulatory elements predicted for these two distant clades heavily overlapped with the elements ascertained for the entire *Tetrapoda *superclass (Table 1), suggesting a remarkable conservation of *cis*-acting splicing regulatory factors in vertebrate evolution.

**Table 1:** Intersection between putative intronic enhancers found separately for primates and outgroup clades and for the entire *Tetrapoda *superclass.

	**Outgroup 5'SS ISEs/ISSs**	**Outgroup 3'SS ISEs/ISSs**	**Vertebrates 5'SS ISEs/ISSs**	**Vertebrates 3'SS ISEs/ISSs**
Primates 5'SS ISEs/ISSs	62/4.96	59/18.84	577/24.73	105/65.14

Primates 3'SS ISEs/ISSs	25/7.68	278/28.46	58/47.67	1,687/130.76

Vertebrates 5'SS ISEs/ISSs	622/33.35	327/130.27	2,428/101.57	297/231.66

Vertebrates 3'SS ISEs/ISSs	127/93.11	3,166/366.87	297/231.66	6,436/480.96

Here is shown the ratio between the actual intersection and the expected intersection of the sets under the null hypothesis (expected intersection between the same number of randomly generated oligos). An intersection between the two sets of elements is calculated as the number of all the possible longest common substrings *LCS *of the two compared elements *a *and *b*, with the size | *LCS*| ≡ min(|*a*|,|*b*|), in ordered pairs (*a*, *b*) coming from the Cartesian product of the sets.

We compared groups of the predicted exonic and intronic enhancers/silencers to better understand the "splicing code" supporting the exon definition. As could be seen in [see Additional File 1 Table S1] groups of ISEs supporting 5'SS and 3'SS sides intersect only half as expected by a random chance. This observation supports a hypothesis that independent mechanisms define neighboring exons and they do not share intronic enhancers located within common introns. On the contrary, ISSs are approximately four times more likely to be shared by the 5'SS and 3'SS sides, compared to a random chance expectation, and seem to play an active role in creating a "silencing" background within introns [21]. The group of 5'SS ISEs has substantial intersection with the 5'SS ESSs. This finding is consistent with previous observations that 5'SS ISEs frequently play silencing role if misplaced within exons [22]. This is further supported by a pronounced antagonism between 5'SS supporting ISEs and ESEs [see Additional File 1 Table S1].

Table 2 puts the motifs we have found in retrospective context of previously reported elements shown in Table 3. Many of these elements were confirmed by the splicing reporter constructs. After excluding previously identified elements [10-12,14-18] from our *Tetrapoda *identified elements, a set of 373 novel oligomers was identified [see Additional File 1 Section 7]. Statistical significance for the motifs found along with LOD scores is shown in [see Additional File 1 Section 8].

**Table 2:** Intersection of predicted elements with the systematically identified elements reported in Table 1.

	**RESCUE- ESEs **[7]	**Wang et al. decamers **[24]	**Yeo et al. 5'SS ISEs 5-mers **[10]	**Yeo et al. 3'SS ISEs 5-mers **[10]	**Zhang et al. PESEs **[8]	**Zhang et al. PESSs **[8]	**Zhang et. al. EIEs **[12]	**Zhang et. al. IIEs **[12]	**Wang et.al. ISEs/ISSs **[14]	**Goren et. al. ESRs **[11]
**5'SS ISEs**			3/9.87		8/202.52		118/330.24	450/206.73	16/83.80	

**5'SS ISSs**		105/9.46				68/54.19			46/27.40	

**5'SS ESEs**	3/2.90				4/5.75		8/13.81	14/8.64		2/3.47

**5'SS ESSs**		4/0.84				0/4.61	38/22.64	19/14.17		4/5.70

**3'SS ISEs**				0/30.64	183/173.03		662/614.92	337/384.94	422/156.04	

**3'SS ISSs**		156/34.31		25/35.42		83/190.50			213/164.59	

**3'SS ESEs**	32/8.89				2/29.87		68/42.25	28/26.45		13/10.65

**3'SS ESSs**		0/0.21				1/1.10	19/12.98	6/8.12		5/3.27

Here is shown the ratio between the actual intersection and the expected intersection of the sets under the null hypothesis (randomly generated oligos). An intersection between the two sets of elements is calculated as the number of all the possible longest common substrings *LCS *of the two compared elements *a *and *b*, with the size | *LCS*| ≡ min(|*a*|, |*b*|), in ordered pairs (*a*, *b*) coming from the Cartesian product of the sets.

**Table 3:** Splicing regulatory elements previously predicted by systematic studies.

**Publication**	**Number of elements predicted**
Fairbrother, W.G., et al. [7]	238 hexamers as candidate ESEs

Zhang, X.H. and L.A. Chasin [8]	Putative 2,069 octamers as exonic splicing enhancers and 974 octamers as exonic splicing silencers

Wang, Z., et al. [24]	133 ESS-containing decanucleotides

Yeo, G.W., E.L. Van Nostrand, and T.Y. Liang [10]	133 5'SS ISEs and 299 3'SS ISEs pentamers

Goren, A., et al. [11]	285 hexamers putative exonic splicing regulatory sequences

Zhang, C., et al. [12]	Putative 1131 hexamers Exon-Identity Elements (EIEs) and 708 Intron-Identity Elements (IIEs)

Stadler, M.B., et al. [13]	380 hexamers as new candidate ESEs and 132 hexamers as new candidate ESSs

Wang, E. T., et. al. [14]	187 5'SS ISEs/ISSs and 175 3'SS ISEs/ISSs hexamers supporting the tissue-specific splicing events

### Higher conservation of intronic elements

A higher evolutionary conservation of the elements found in the proximity of exonic flanks compared to the background sequences would be an important indicator of their functional importance in splicing regulation [10,14]. Within 12,000 multiple intronic flank sequence alignments we found a significantly higher conservation of the predicted intronic *cis-*acting octamers as compared to all other possible motifs (Table 4). Here we considered only the predicted octamers for uniform estimates of conservation scores, which would not be possible for elements of different sizes. Conservation degrees of ISSs and ISEs shown in Table 4 are similar, which suggests the importance of both enhancers and silencers in splicing definition.

**Table 4:** Counting number of conserved octamers in the exonic proximity

**Intronic flanks next to 5'SS**			**Intronic flanks next to 3'SS**		
**5'SS ISEs**			**3'SS ISEs**		

	**Conserved**	**Non-Conserved**		**Conserved**	**Non-Conserved**

Elements	4,024	6,800	Elements	4,272	8,363

Non- elements	251,842	518,369	Non- elements	261,387	578,106

Fisher 2-tail test: 1.81 × 10^-22^			Fisher 2-tail test: 1.59 × 10^-10^		

**5'SS ISSs**			**3'SS ISSs**		

	**Conserved**	**Non- Conserved**		**Conserved**	**Non- Conserved**

Element	399	648	Element	6,537	11,385

Non- elements	251,842	518,369	Non- elements	261,387	578,106

Fisher 2-tail test: 0.00025			Fisher 2-tail test: 3.46 × 10^-51^		

Set of all other possible elements was obtained by excluding the ISEs and ISSs supporting either 5' or 3' SSs from the set of all possible octamers. We counted cases where oligonucleotides stay entirely conserved versus changing in at least one nucleotide position between the pairs of sequences from multiple sequence alignments, where only the motifs containing no gaps were considered. In case of 5'SS elements we considered window of size 20 nt starting 16 nt downstream from 5' exonic boundary in human sequence, where in case of elements supporting 3'SS we considered 20 nt window ending 63 nt upstream of 3' exonic boundary.

### Secondary structure association with the elements

**Figure 1:**
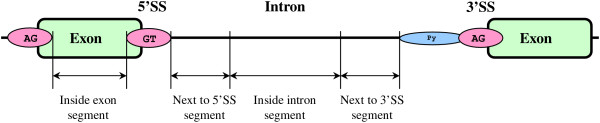
**Four segments for testing PU values for the predicted elements**. Next to the 5'SS and 3'SS segments were chosen to extend 50 nt context, not including ± 30 nt, inside intron from the corresponding exonic boundaries.

According to [2] splicing enhancers and silencers are preferentially located in a single stranded region of RNA as compared to the controls, especially in the vicinity of SSs. This has been explained by the higher probability of trans-acting factors, such as SR proteins, to bind local single-stranded regions. We therefore determined the Probability Unpaired (PU) values for the predicted elements [see subsection *Statistical analysis*]. We considered only predicted octamers to obtain the PU values on the same scale which would be problematic for elements of different sizes. We examined sequences composed of predicted octamers surrounded with ± 30 nt context located in various segments of exons and introns as shown in Figure 1. PU values are known to be strongly associate with GC content of the motifs and the surrounding context [2], therefore it would be most informative to evaluate the difference in the distribution of PU values for the same group of elements surrounded by wild type and dinucleotide reshuffled contexts. Table 5 presents the average PU values for the elements located in the different segments before and after reshuffling.

**Table 5:** Average PU for the predicted octamer elements surrounded by ± 30 nt context analyzed in various segments as shown in Figure 2.

**Elements**	**Next to 5'SS**		**Next to 3'SS**		**Inside intron**		**Inside exon**	
	
	**Number**	**Average PU**	**Number**	**Average PU**	**Number**	**Average PU**	**Number**	**Average PU**
**5'SS ISEs**	3,946	0.100/0.100	3,954	0.185/0.191	4,014	0.189/0.196	4,017	0.148/0.154

**5'SS ISSs**	1,361	0.174/0.184	700	0.241/0.247	3,993	0.182/0.173 **(P = 0.0064)**	1,744	0.157/0.144

**3'SS ISEs**	3,930	0.165/0.165	4,061	0.205/0.206	3,989	0.190/0.187	3,984	0.182/0.174

**3'SS ISSs**	3,954	0.144/0.163	3,987	0.120/0.127	4,039	0.194/0.185 **(P = 0.0063)**	4,050	0.133/0.132

**All Other elements**	4,004	0.128/0.130	3,966	0.163/0.160	4,053	0.152/0.146 **(P = 0.0083)**	4,024	0.134/0.123

We classified the predicted elements surrounded by ± 30 nt context according to segments of their location. The mean PU values calculated according to [2] for wild type and dinucleotide reshuffled contexts are followed by significant P-values obtained with the Wilcoxon two-sided rank-sum tests. Only the P-values rejecting the null hypotheses (P < 1%) that the distribution is similar in the two groups of PU values are shown.

Having the numerical series of PU values in various segments for different types of elements, we estimated if their distribution changes after dinucleotide reshuffling with the two-sided Wilcoxon rank-sum test as shown in Table 5. Our working hypothesis was that if predicted enhancers/silencers are preferentially supported by a single-stranded configuration then average PU values should go down after contextual reshuffling as it would most probably disrupt the naturally occurring local secondary structures. We did not find statistically significant discrepancies in the distribution of PU values after reshuffling the contexts of elements located in segments associated with SS regulatory functions ('Next to 5'SS', 'Next to 3'SS' and 'Inside exon') as shown in Figure 1. The only exception was the insignificant reduction of PU values for both 5' and 3' ISSs located in deep intronic segments as could be seen in Table 5. This statistical significance is highly reproducible and holds even for reduced size subsets of 600 ISSs examined deep inside intron (P = 0.0072 for 5'SS ISSs and P = 0.027 for 3'SS ISSs).

### Implication of elements found in splicing reporter experiments

In order to investigate the implications of elements found in splicing regulation, we considered systematic mutation experiments presented in [23] (Figures Eight, Nine). The results of these experiments are interpreted through the mutation induced changes in the predicted 3'SS regulatory elements [see Additional File 1 Table S3]. Original experimental design [23] considered the influence of exonic silencers on selection of competing 3'SSs in human gene coding for proinsulin (INS) and hepatic lipase (LIPC). Here we noticed that according to [see Additional File 1 Table S1] predicted 3'SS ISSs are three times more likely to overlap with 3'SS ESSs compared to overlap by random chance, which indicates that most of the 3'SS ISSs elements also act as 3'SS ESSs. This is further supported by noticing that FAS-ESS elements AGGGGT and GGAGGG [9] are similar to our predicted 3'SS ISSs GGAGGGG (A.IE -2.00) and TGGAGGG (A.IE -2.08) and a substantial overlap between predicted 3'SS ISSs and FAS-ESS decamers [24] as could be seen in Table 3. As could be seen in [see Additional File 1 Table S3] removal of our 3'SS ISSs generally results in increased inclusion of isoform 4 (rows 4 ⇒ 5, 12 ⇒ 13, 14 ⇒ 15) and newly introduced 3'SS ISSs result in increased inclusion of isoform 3 (rows 3 ⇒ 4, 6 ⇒ 7, 11 ⇒ 12). Same tendency is observed in [see Additional File 1 Table S4], where removal of 3'SS ISSs increases level of IVS-78 isoform inclusion (rows LIPC -WT ⇒ ESS - 1, ESS - 3 ⇒ ESS - 4, ESS - 6 ⇒ ESS - 7 and ESS - 10 ⇒ ESS - 11) newly introduced 3'SS ISSs result in an opposite effect (rows ESS - 2 ⇒ ESS - 3, ESS - 5 ⇒ ESS - 6, ESS - 9 ⇒ ESS - 10). Introduction of 3'SS ESE signal TAGGTC (A.EE 1.72) results in increased IVS-78 isoform inclusion as expected (row ESS - 13 ⇒ ESS - 14). These findings suggest an active role of the predicted elements in SSs regulation.

### Comparison of newly identified elements with known binding sites for RNA binding proteins

To further support the functional importance of the predicted elements we compared elements found with the oligonucleotides already known to attract RNA binding factors actively involved in splicing.

CA repeats bound by hnRNP L [25] are located in clusters D.IE.14 and A.IE.9 (here and further in this section we refer to [see Additional File 1 Section 4] listing the clusters of elements predicted). Clusters D.IE.4, A.IE.10, A.IE.12 are enriched with elements YCAY that bind the NOVA family of neuron specific splicing factors [22]. Poly-G signal has been reported simultaneously as an ISE signal [26] when located downstream of a 5' splice site (clusters D.IE.6, D.IE.12 and D.IE.15 are enriched with these elements) and play a role of an exonic silencer (cluster A.EE.5) when located inside exon [22]. The G-run-binding factor hnRNP H is known to participate in exon definition [27,28]. Compact cluster A.EE.3 contains hnRNP A1 SELEX predicted binding domain TAGGTC [27] and clusters A.IE.7 and A.IE.4 contain hnRNP A1 binding elements TAGGG(A/T) [27]. Clusters A.IE.6 and A.IE.7 contain elements AGGAGGA, CAGAGGA, CAGAGGG that were identified by SELEX procedure as binding targets for SF2/ASF enhancer [28]. Clusters A.IE.8 and A.IE.12 are enriched with consensus binding motif ACTAAC of STAR family RNA-binding factors, in particular quaking homologue (QKI) [29].

Elements TGTGT and TGTT were established as active cores of primary binding sites of ETR-3 splicing regulator after five rounds of SELEX procedure [30] where many clusters, such as A.IE.8 and A.IE.15, are enriched with such elements. From AEDB database [31] 77 motifs were selected known to influence splicing in their natural context [2], many of these elements are similar to our predicted elements. We have identified 42 out of 71 confirmed splicing modulating motifs of size greater than 4 nt to intersect with our predicted elements as shown in [see Additional File 1 Table S2].

## Conclusion

Using the orthologous exons currently available for 23 *Tetrapoda *organisms we have identified 2,546 unique splicing regulatory elements. Among these elements 203 (7.97%) 3'SS and 177 (6.95%) 5'SS supporting motifs are novel and have not been previously reported in systematic screens detecting such elements. Among our predicted elements, 51.81% were octamers and 41.08% of sequences were heptamers as compared to only 6.76% hexamers and 0.35% pentamers, suggesting that motifs of larger size play important role in splicing regulation. We detected intersections with some of the *cis*-acting elements reported in the previous studies, but not nearly as dramatic as we saw between the intronic elements predicted for primates and *Tetrapoda *non-eutherian (an outgroup) clades. It demonstrates high reproducibility of our results obtained for various vertebrate lineages and supports the existence of highly conserved splicing regulatory code across vertebrates. This result also suggests the implications of elements found in regulating human splicing and may help explaining human hereditary disorders caused by mutations modulating such elements. We have established the higher evolutionary conservation for the predicted intronic *cis*-acting elements within mammalian intronic flanks which indicates their functional significance in exon definition. The elements found contain many of the known *cis*-acting factor binding sites with functionality supported by experiments with splicing reporter constructs. All these lines of evidence suggest active involvements of the predicted elements in control gradient directing spliceosome to the proper exons in the process of pre-mRNA splicing [23].

We did not observe statistically significant association for the predicted groups of *cis*-acting elements with the secondary pre-mRNA local structure in the vicinity of the SSs, except for slightly increased single strandedness detected for 5' and 3' ISSs deep inside introns. This observation is in contrast to the earlier reported [2], where known splicing regulatory motifs were identified as more single stranded compared to controls in exonic vicinity. Our result may indicate a potential mechanism of how ISSs-mediated silencing background keeps spliceosomal components inactive in the deep intronic sequences by providing stronger than normal binding affinity to preferentially single-stranded ISSs.

A remarkable intersection between the 5'SS ISSs and the 5'SS ESSs [see Additional File 1 Table S1] is explained by the highly improbable chances of having elements containing a core fragment of a strong 5'SS competitor consensus in vicinity of a 5'SS. We have also established that many 3'SS ISSs act as 3'SS ESSs. These observations suggest that discovered splicing regulatory elements have broad functionality spectrum spreading beyond genomic segments where they have been originally found, such as possible regulatory role in 3'UTR [14].

## Methods

### Collection and validation of *Tetrapoda *exons

We parsed and extended blocks of orthologous with human reference exons from multiple sequence alignment of 17 vertebrate genomes obtained from UCSC genome browser [32]. The following tetrapods were processed: Human (*Homo sapiens*), Chimpanzee (*Pan troglodytes*), Rhesus (*Macaca mulatta*), Mouse (*Mus musculus*), Rat (*Rattus norvegicus*), Rabbit (*Oryctolagus cuniculus*), Dog (*Canis familiaris*), Cow (*Bos taurus*), Armadillo (*Dasypus novemcinctus*), Elephant (*Loxodonta africana*), Tenrec (*Echinops telfairi*), Opossum (*Monodelphis domestica*), Chicken (*Gallus gallus*), Frog (*Xenopus tropicalis*). The "threaded blockset alignments" [33], built under the assumption that all matching segments occur in the same order and orientation in the given sequences, were projected onto human reference exons predicted by the spliced alignment of human reference sequences  against reference human chromosomal assemblies  using the BLAT program [34]. Having the chromosomal sequences of corresponding organisms, the blocks from the multiple genome alignments were extended to include splicing signals and 205 nt intronic flanks. We collected functionally important regions of intronic flanks normally located no further than 100 nt from the exons [14,35] and deep intronic sequences which we used as background model located beyond 100 nt from the SSs. The splicing signals flanking the extended exons have been double checked with the Bayesian SSs sensor [36] to make sure the extension yielded the correct exonic boundaries and the splicing signals flanking the exons have statistically significant score indicating their splicing competence. We kept only one isoform per gene with the largest number of predicted exons.

This exon set was extended with exons derived from processing of 28 vertebrates multiple genome alignments obtained from UCSC genome browser [37] from the following tetrapods: Bush Baby (*Otolemur garnetti*), Tree Shrew (*Tupaia belangeri*), Guinea Pig (*Cavia porcellus*), Shrew (Sorex araneus), Hedgehog (*Erinaceus europaeus*), Cat (*Felis catus*), Horse (*Equus caballus*), Platypus (*Ornithorhynchus anatinus*), Lizard (*Anolis carolinensis*). The blocks from a total of 28 vertebrates multiple genome alignments are normally shorter than blocks from 17 vertebrates multiple genome alignments, therefore chances are higher that the block extension may not produce the correct exonic boundaries. Only the exons associated with the species not obtained through the first round should be processed in the second round.

To establish the firm ground for using sequences from distantly related organisms in predicting common SS proximal elements and to estimate implication of elements found in modeling human splicing we conducted independent search for the elements in two distantly related clades of primates and non-eutherian *Tetrapoda *organisms. For these purposes we have examined 489,668 extended exons in primates clade (Human (*Homo sapiens*), Chimpanzee (*Pan troglodytes*), Rhesus (*Macaca mulatta*), Bush Baby (*Otolemur garnetti*)) and 476,218 extended exons from non-eutherian *Tetrapoda *organisms (Opossum (*Monodelphis domestica*), Chicken (*Gallus gallus*), Frog (*Xenopus tropicalis*), Platypus (*Ornithorhynchus anatinus*), Lizard (*Anolis carolinensis*)) taken as the most distant outgroup (a group of species known to be phylogenetically outside the primates clade) among *Tetrapoda *organisms relative to primates.

To estimate the increased conservation of the intronic elements found within the intronic flanks we have used the Prank [37] tool to built multiple sequence alignments of the orthologous intronic flanks (12,000 for 5' and 3' sides) including primates (Human (*Homo sapiens*), Chimpanzee (*Pan troglodytes*), Rhesus (*Macaca mulatta*), Bush Baby (*Otolemur garnetti*)) and rodents (Mouse (*Mus musculus*), Rat (*Rattus norvegicus*), Guinea Pig (*Cavia porcellus*)) clades.

Through the literature search we collected the test set of 185 human genes previously linked to autism spectrum disorder and genes implied in environmental response [38]. A set of extended exons obtained through the spliced alignment of human reference sequences for the test set against the reference chromosomal assemblies, as described previously, was used as a sample representative collection of important human genomic regions with potential implication in medical practice. The set included 4,650 canonical 5' and 3' SSs flanking internal exons and was used to estimate association of local pre-mRNA secondary structures with the predicted elements.

### Statistical analysis

**Figure 2:**
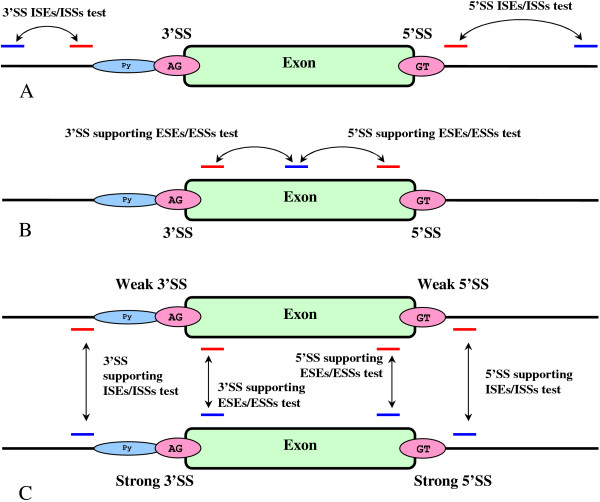
**Location of genomic regions used for comparative analysis**. (A) Statistical significance tests for intronic enhancing/silencing elements surrounding exon. Blue is the null-hypothesis region and red is the region of statistical significance associated with the exon proximity. The red region is specifically located outside the area associated with donor or acceptor signal consensuses [36]. (B) Statistical significance test for the ESEs/ESSs elements supporting the exonic definition. This strategy allows canceling the statistical biases associated with the protein coding potential best characterized by the hexamer statistics [41] and focusing at the essential difference between the exonic flanks, normally enriched with ESEs [42], and the middle section supposedly depleted of such elements. (C) The differential strategy allows detecting enhancing and silencing elements that have substantially different concentration in vicinity of a strong vs. weak SS as defined by the Bayesian SS sensor [36]. The score from the sensor is measured on a discrete scale from 1 to 5, where 1 stands for a weak signal and 5 stands for strong.

**Figure 3:**
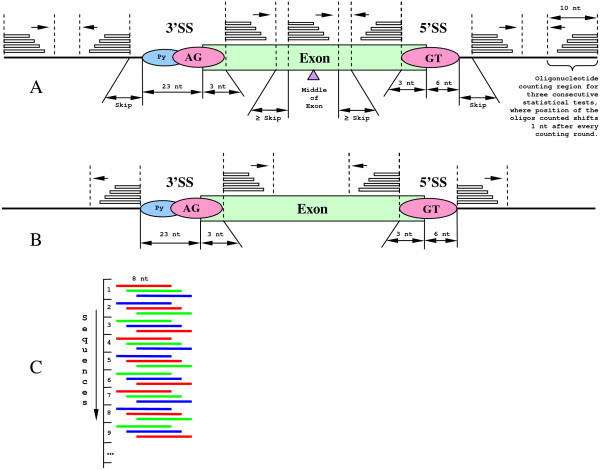
**Location of the counting regions used for oligonucleotide scoring relative to exonic flanks**. All short exons that were not able to accommodate the regions are disregarded. (A) The region arrangement for the counting strategies shown in Figures 2 (A) and (B), where the *Skip *value is set to 0 nt for the first comparative measurement and 29 nt for the second. The second comparative measurement is necessary to predict active intronic elements that have maximum enhancing/silencing potential at certain optimal distance from the exonic boundary, such as polyG signals [26]. The second measurement also trades the smaller number of longer exons considered for the greater chance of detecting element density discrepancy between the middle of the exons and the flanks. (B) The region arrangement corresponding to differential test strategy shown in Figure 2 (C). (C) The tiling strategy within a region increases the variety of elements sampled in a counting round. Tree different colors used to show which oligo within a region gets sampled in a three consecutive statistical tests (red in the first test, green in the second test, blue in the third test). This strategy reduces chances for multiple sampling of the same oligo conserved at a certain position in closely related organisms.

We measured a statistically significant bias for all the possible oligonucleotides of size equal or less than 8 bp in the vicinity of true 5' and 3' SSs compared to distant "background" locations as shown in Figures 2 and 3. Our assumption is that enhancers are more common and silencers are less frequent in vicinity of SSs compared to "background". For the convenience of representation all the elements were scored using prefix tree structure as shown in [see Additional File 1 Figure S1] to determine statistical significance. The tree structure of height 8 has 65,536 leaves associated with any possible octamer where each internal node and root have out-degree 4 corresponding to the number of possible nucleotides at a next position. When octamers get inserted in the tree the counts associated with the traversed internal nodes and the destination leaf node increase. The scores associated with an internal node of certain *depth *correspond to the density of an oligonucleotide of size *depth *present at a certain positions within genome. A significant deviation of an oligo density in the proximity of SSs as compare to background locations is strongly indicative of important functionality of elements related to splicing [10,15,33].

Comparing oligo counts at the significance level of *α *= 0.011 using *χ*^2 ^test involved 87,380 statistical hypotheses testing for all possible oligos of size less or equal to 8 bp located at certain position relative to a SS. Following the Bonferroni correction for multiple hypothesis testing we reduced the significance level to  for individual tests. All the *χ*^2 ^tests for the *SS vicinity counts *≥ 63 or *deep intronic/exonic counts *≥ 63 are statistically significant



under conditions



or



Comparative measurements between the regions shown in Figure 3 were made in 3 rounds according to experimental schemas shown in Figure 2. Every round of scoring involved all the sequences from the exonic set, elements predicted in any of these 3 scoring rounds were reported. The second comparative measurement for the *Skip *value 29 nt, as shown in Figure 3(A), was necessary to detect intronic enhancers/silencers that have maximum impact on splicing when located at certain optimal distance from the exonic flanks, which is the known fact in case of polyG signals [26]. Elements detected in the first comparative measurement (for *Skip *= 0 nt in Figure 3(A)), in the second measurement (for *Skip *= 29 nt in Figure 3(A)) and for the third differential measurement as shown in Figures 2(C) and 3(B)) were merged in one prediction.

Extended orthologous exons associated with the same multiple genome alignment block frequently contain identical conserved oligonucleotides at certain positions relative to SSs, especially within exons. These conserved elements violate our assumption of independence of the sequences used for analysis of statistical significance; therefore within a block we counted unique element at certain position relative to SSs only once, disregarding all other identical motifs conserved since speciation from a common ancestor. To prevent substantial element underscoring in a counting round, since many of the elements at a certain position relative to a SS were sorted out due to evolutionary conservation, we shifted an element position by one within a counting region for each consecutive sequence as shown in Figure 3(C), where element coordinate within a region were calculated according to formula



where mod is a modulo operation, counting round could be 0,1 or 2 and the sequence index goes from 1 to 2,333,379. Elements shifted by one position within the region are normally different and therefore not sorted out for being similar, which allows combining more elements in the region associated with a block under the same evolutionary pressure.

These predicted groups of elements were clustered with the Mixture of Hidden Markov Models (MHMM), an unsupervised clustering method capable of modeling dependencies between neighboring positions in active motif cores [see Additional File 1 Section 3].

The PU values for the predicted octamers surrounded by ± 30 nt context were calculated as described in [39] using RNAfold [40] program from Vienna RNA package . To accelerate finding average PU value for an element we calculated them only for the contexts of 11, 15, 20, 25 and 30 nt according to [2], a method which produced consistent results for perfect loop configuration (PU = 1), perfect stem configuration (PU = 0) and a very similar PU value for the example in [2] (Figure 1) for natural pre-mRNA structure supporting TCTCTCT element. We have also confirmed that 77 known enhancer/silencer elements are more single stranded since average PU values were going down after dinucleotide contextual reshuffling (the control) as reported in [2]. The dinucleotide reshuffling procedure [41] were making 10,000 iterations equally distributed between the non-overlapping dinucleotides swapping within or across flanking segments, excluding the elements. This way we kept the same GC content which is essential for proper PU values comparison in case/control studies [2].

## Authors' contributions

AC designed and implemented the framework to search for splicing regulatory elements and tested the application. CH initiated the study, provided many valuable suggestions steering the project and was the head of the lab where the work was done. IV provided necessary expertise to formulate the study objectives, obtained experimental data to support newly found elements and extensively edited the manuscript. All authors read and approved the final manuscript.

## Supplementary Material

Additional file 1 **Report and analysis of splicing regulatory elements found**. Contains list of all the elements found along with their statistical significance and overlap between their groups. Functional analysis for these elements in context of alternative splicing constructs is reported. We provided brief description for an unsupervised clustering method used to group the elements found and grouped the elements within possible functional motifs. We also report elements found for primates and outgroup. Subset of novel elements is reported. Click here for file
